# The Possible Role of Microorganisms in Mosquito Mass Rearing

**DOI:** 10.3390/insects12070645

**Published:** 2021-07-15

**Authors:** Luca Chersoni, Alice Checcucci, Marco Malfacini, Arianna Puggioli, Fabrizio Balestrino, Marco Carrieri, Irene Piunti, Maria Luisa Dindo, Paola Mattarelli, Romeo Bellini

**Affiliations:** 1 Centro Agricoltura Ambiente “G. Nicoli”, IAEA Collaborating Center, Sanitary Entomology and Zoology Department, Via Sant’Agata 835, 40014 Crevalcore, Italy; mmalfacini@caa.it (M.M.); apuggioli@caa.it (A.P.); fbalestrino@caa.it (F.B.); mcarrieri@caa.it (M.C.); piunti.irene@gmail.com (I.P.); rbellini@caa.it (R.B.); 2 Department of Agricultural and Food Science, University of Bologna, Viale G. Fanin 42, 40127 Bologna, Italy; alice.checcucci2@unibo.it (A.C.); marialuisa.dindo@unibo.it (M.L.D.); paola.mattarelli@unibo.it (P.M.)

**Keywords:** *Aedes albopictus*, SIT, mass-rearing, microbiota, probiotics, biological control

## Abstract

**Simple Summary:**

One of the most promising control methods against *Aedes albopictus* is the sterile insect technique, which consists of mass rearing the target species, separation of males from females, and male exposure to sterilizing ionizing radiation. Once released in the environment, the sterile males are expected to search for wild females to mate with. The quality of sterile males is a crucial aspect in SIT programs in order to optimize effectiveness and limit production costs. The integration of probiotic microorganisms in larval and adult mosquito diets could enhance the quality parameters of the released sterile males.

**Abstract:**

In Europe, one of the most significant mosquitoes of public health importance is *Aedes albopictus* (Skuse), an allochthonous species of Asian origin. One of the most promising control methods against Aedes albopictus is the sterile insect technique (SIT), which consists of mass rearing the target species, separation of males from females, and male exposure to sterilizing ionizing radiation. Once released in the environment, the sterile males are expected to search for wild females to mate with. If mating occurs, no offspring is produced. The quality of sterile males is a crucial aspect in SIT programs in order to optimize effectiveness and limit production costs. The integration of probiotic microorganisms in larval and adult mosquito diets could enhance the quality parameters of the released sterile males. In this review, we attempt to give the most representative picture of the present knowledge on the relationships between gut microbiota of mosquitoes and the natural or artificial larval diet. Furthermore, the possible use of probiotic microorganisms for mosquito larvae rearing is explored. Based on the limited amount of data found in the literature, we hypothesize that a better understanding of the interaction between mosquitoes and their microbiota may bring significant improvements in mosquito mass rearing for SIT purposes.

## 1. Introduction

*Aedes*, *Culex*, and *Anopheles* mosquitoes can be considered among the most relevant, medically important insects responsible for spreading human diseases, which are either caused by parasites (e.g., malaria and filariasis) or by numerous arboviruses (e.g., Dengue, Chikungunya, Zika, and West Nile) [1]. Nowadays, the diseases transmitted by these arthropods show high mortality and morbidity, leading to health emergencies in many countries. In 2018, for example, over 228 million cases (including 405,000 deaths) of malaria were reported, of which 212 million occurred in Africa (380,000 deaths) [2].

**Figure 1:**
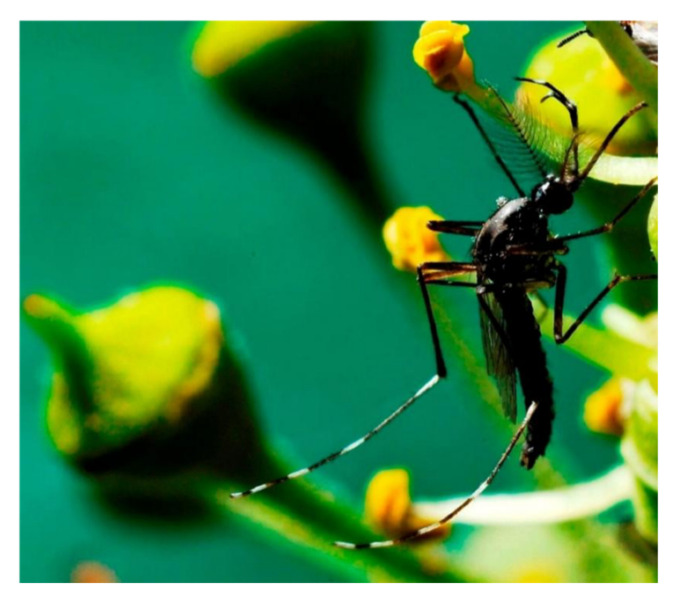
Adult male of *Aedes albopictus* [16].

In Europe, one of the most significant mosquitoes of public health importance is *Aedes albopictus* (Skuse), an allochthonous species of Asian origin, which has colonized the urban areas in many countries (Figure 1) [3]. One of the reasons for the large impact of this mosquito species is linked to its vectorial capacity for 22 different arboviruses, including dengue, yellow fever, and chikungunya [4]. Despite its tropical origin, *Ae. albopictus* has been shown to transmit viruses causing epidemics in temperate regions, as was the case for chikungunya in Emilia-Romagna (Italy) in 2007 and in Lazio and Calabria (Italy) in 2017 [5,6].

The control methods used against *Ae. albopictus*, such as larval control, breeding site elimination, and involvement of residents, produce insufficient levels of population reduction [7,8]. Therefore, different approaches employing genetically modified or Wolbachia-infected mosquitoes have been proposed as a possible tool for the control of mosquitoes and mosquito-borne diseases. Currently, one of the most promising approaches is the sterile insect technique (SIT), which consists of mass rearing of target species, separation of males from females, and male exposure to sterilizing ionizing radiation. Once released in the environment, the sterile males are expected to search for wild females to mate with. If mating occurs, no offspring is produced. Repeated releases lead to a progressive decline in the wild population [9]. The continuous release of a large number of competitive sterile males is therefore crucial. Consequently, it is necessary to create large facilities for the production of huge numbers of high-quality sterile males that can compete with wild males [10,11,12,13]. The quality of sterile males is a crucial aspect in SIT programs and must be accurately considered in order to optimize effectiveness and limit production costs. From a performance perspective, producing better quality sterile males can lead to the release of more competitive insects in the field. This aspect can be very useful, as it could result in a smaller number of released males, thus reducing production costs [14,15]. The achievement of this objective could be facilitated by the standardization and improvement of the rearing conditions, including the optimization of the larval medium.

Mosquito larvae feed on different organic particles, which may be either suspended or settled in water, including a wide range of organisms, viz.: bacteria, fungi, protozoa, algae, and small invertebrates [17,18,19,20]. Food consumed during the larval period has been shown to influence larval development and, consequently, the course of adult life [21,22].

Insects possess a complex microbiome, which is the collection of microorganisms colonizing the body and their genetic material. The relationship between organisms and their microbiomes is very strict, and although microbes inhabit multiple parts of the body (buccal cavity, external parts, etc.), most of what is known about the microbiome focuses on the gastrointestinal tract [23]. Hosts benefit from complementing the functions encoded in their own genomes with those of their associated microbiomes. In many insects, the microbiome plays a fundamental role in many physiological processes and in host health and diseases [23]. Microbiota is strictly linked to diet, environmental factors, and genetics of the host.

Therefore, the interaction between larvae, diet, and the microbiota represents a tripartite pattern that can be shaped properly to obtain the best mass rearing condition in terms of mosquito production and competitiveness of sterilized males.

As already proposed for the implementation of SIT mass rearing facilities of important agronomic pests [24], the integration of probiotic microorganisms in larval and adult mosquito diets could enhance the quality parameters of the released sterile males. Our aim here is to review the current knowledge on bacteria, fungi, and viruses present in the gut microbiota of mosquitoes and their relationships with natural and artificial larval diets. The innovative use of probiotic microorganisms for mosquito larvae rearing is explored, with the effects on mosquito development and overall quality at immature and adult stages highlighted.

Although there are many factors that can interfere with larval development, such as water temperature, water quality, and environmental conditions, in this review, we focus exclusively on the microorganisms that can act as supplements to the microbiota, in order to improve various results of mosquito breeding, such as development and larval growth and the performance of the irradiated adult mosquitoes. For the same reason, this review does not investigate the aspect linked to microorganisms that can be pathogens or control agents of mosquito larvae.

## 2. Mosquito Microbiota and Environmental Factors

The microbiota plays a fundamental role in mosquito development and survival [25,26,27], promoting the synthesis of essential substances such as vitamins, amino acids, carbohydrates, and steroids [28]. Furthermore, it has been demonstrated that the mosquito microbiota can shape the host fitness and modulate the insect immune system, including the vector competence [29,30]. Digestion and detoxification are results of different interactions between the mosquito and the symbiotic microorganisms that can influence the mosquito development and adult nutritional reserves [28]. Environmental and genetic factors, such as the location of mosquito collection [31,32], the mosquito species [33], and the rearing conditions [34], are considered important drivers that shape gut microbiota composition.

### 2.1. Bacteria

It has been observed that microbiota composition is strictly linked to the stages of mosquito development. Recent research showed that Actinobacteria, Proteobacteria, and Bacteroidetes phyla are always present in the 4th instar larvae of *Ae. albopictus* and *Ae. aegypti* (Linnaeus). Within these phyla, there are *Chryseobacterium*, *Elizabethkingia*, *Pseudomonas*, *Neisseria*, and *Enterobacter* [35]. Some genera of Actinobacteria, such as *Leucobacter* and *Microbacterium*, were only found in larvae, while the genus *Chryseobacterium* was detected in all life stages, including the adult stage [25].

Scolari et al., reported that more than 90% of the intestinal microbiota of *Ae. aegypti* larvae is lost during metamorphosis [35]. By analyzing nine mosquito species, a recent work by Mancini et al., highlighted that Proteobacteria, Bacteroides, Firmicutes, and Actinobacteria represent more than 99% of the total microbiota community components [36]. More specifically, the most frequently described bacteria in the gut of adult *Aedes* spp. are members of Enterobacteriaceae (e.g., *Enterobacter, Klebsiella, Kluyvera*), Erwiniaceae (e.g., *Pantoea*), Yersiniaceae (e.g., *Serratia*), Acetobacteraceae (e.g., *Asaia*), Enterococcaceae (e.g., *Enterococcus*), and Bacillaceae (e.g., *Bacillus*) [35].

In a more recent study, Scolari et al. [37] investigated on the composition of the bacterial microbiota in *Ae. albopictus* at larval and adult stages. In addition, the researchers defined the bacterial composition in water from a natural breeding site, focusing on the difference of bacterial diversity before and after the adult’s emergence.

The natural environment is important in determining the composition of mosquito microbiota. It was observed that the presence of artificial substances, such as fertilizers or antibiotics, could shape the microbial composition of the environment itself, changing mosquito larval development [38]. Modification in *Anopheles gambiae* Giles microbiota, following interaction with antibiotic residues in human blood, made mosquitoes more sensitive to *Plasmodium* spp. [39]. Indeed, Guégan et al., observed a reduction, up to elimination of the bacterial taxa, in the microbiota of *Ae. albopictus* adults following the ingestion of antibiotics and, at the same time, an increase in symbionts such as *Wolbachia* and *Dysgonomonas* [40]. *Dysgonomonas*, a symbiotic bacterium frequently associated with *Ae. albopictus*, is known for the production of vitamin B12 in termites. It is still unknown if it plays a similar role in *Ae. albopictus* [32].

Yadav et al., reported a strong similarity in the bacterial microbiota composition between *Ae. aegypti* and *Ae. albopictus* females living in the same area in northeast India [41].

Wang et al., noticed that mosquitoes were constantly in contact with microorganisms throughout their life cycle, especially during the larval stage, when the aquatic environment hosting great bacterial diversity plays a major role. Wang and colleagues showed that, following the filtration of water in which *Ae. albopictus* larvae were grown, the bacterial content dramatically dropped, causing a decline in adult emergence from 11.0% to 3.3%. Moreover, when water was treated with ampicillin, the percentage of pupation dropped to 0% [42].

These results are in line with the findings of Chouaia et al., who showed that there was a delay in the normal development of *Anopheles stephensi* Liston larvae treated with rifampicin, compared to the untreated groups. In contrast, larvae treated with rifampicin but integrated with a rifampicin-resistant mutant strain of *Asaia* sp. showed development comparable with that of larvae not exposed to the antibiotic [43]. Coon et al., showed that, despite the availability of an adequate diet, *Ae. aegypti* and *An. gambiae* larvae kept without microbiota were unable to develop beyond the first stage. If *Ae. aegypti* larvae containing microbiota rich in *E. coli* were provided with an aseptic diet, they could develop successfully to the adult stage [26,27]. Bacteria present in larval breeding water can be maintained until the larvae emerge from the pupal stage to become adult mosquitoes, as shown by Lindh et al., for *An. gambiae*. At the same time, adult mosquitoes can transmit symbionts in breeding water, thus influencing the microbial composition of the aquatic environment [32,44].

Since the beginning of the 20th century, researchers have been trying to understand what role microorganisms play in the hatching of mosquito eggs. For example, Bacot observed that disinfected eggs of *Ae. aegypti* introduced into a sterile medium could hatch if subjected to different stimuli, with the most effective stimuli provided by live yeast and bacteria [45]. Subsequent studies showed how a reduction in dissolved oxygen in water by chemical or biological factors affected the hatching of *Aedes* eggs [46,47,48], reaching the conclusion that the concentration of dissolved oxygen in water is the main driver of egg hatching. The lowering of the oxygen concentration may depend on the presence of aerobic bacteria in the medium. These bacteria need oxygen in order to carry out their metabolic functions.

Although there is agreement in this effect of dissolved oxygen in water on egg hatch, a study by Ponnusamy et al., showed how a mix of 14 bacterial species isolated from an infusion of *Quercus alba* leaves suspended in a sterile saline solution led to a high rate of hatching of *Ae. aegypti* eggs. In a sterile saline solution, the metabolic activity of bacteria is often very low, leading to high concentrations of dissolved oxygen in the solution. The researchers observed that, as the bacterial concentration in the sterile medium increased, the rate of hatching of the eggs also increased, regardless of the high concentration of the dissolved oxygen. The results showed that, with a bacterial concentration of 10^9^ cells/mL, there was a hatching rate of 93.8% after four hours [49].

Researchers have also focused on the role of microbiota in female oviposition behavior. In a study, Arbaoui et al., observed that *Ae. aegypti* preferred to lay eggs in bamboo infusions from *Bambusa* spp. than in distilled water. The authors determined whether infusion’s microbial cells were mandatory to elicit this effect by filtrating them with 0.45 um pores. They did not observe any differences between the number of eggs laid on filtrated or non-filtrated infusions. This suggests that microbial cells that are usually larger than 0.45 um are not directly responsible for this response. Instead, compounds eventually produced by these microbes can be important for the preference of one site over the other for ovoposition [50]. The preference for sites with conspecifics was greater than other factors, such as the abundance of food for the larvae and the absence of larval predators. This trend had previously been observed in the laboratory as well [51].

Benzon and Apperson have identified two dominant bacterial species that influence oviposition, namely *Acinetobacter calcoaceticus* and *Enterobacter cloacae*. By comparing two of them isolated, they observed that *A. calcoaceticus* induced significantly higher oviposition than *E. cloacae* [52]. In a more recent study, Ponnusamy et al., described the behavioral responses of *Ae. albopictus* and *Ae. aegypti* gravid females at four different densities (10^6^ cells/mL, 10^7^ cells/mL, 10^8^ cells/mL, 10^9^ cells/mL) of a mix of 14 bacterial species and at three different densities (10^6^ cells/mL, 10^7^ cells/mL, 10^8^ cells/mL) of isolated species obtained from an infusion of bamboo (*Arundinaria gigantea*) [53]. They observed different responses between the two mosquitoes depending not only on the bacterial density but also on the bacterial isolate present in the medium. In particular, with the mix of 14 bacterial species, both *Ae. albopictus* and *Ae. aegypti* were more attracted by the medium containing 10^7^ and 10^8^ cells/mL than the control medium but were not attracted by the 10^9^ cells/mL medium. Observing the effects of individual bacterial species, Ponnusamy et al., noticed that *Ae. aegypti* was significantly attracted by seven bacterial species (*Bacillus thuringiensis*, *Enterobacter asburiae*, *Enterobacter cancerogenus*, *Lactococcus lactis*, *Shigella dysenteriae*, *Citrobacter freundii*, and *Brevundimonas vesicularis*) at 10^6^ cells and 10^7^ cells/mL, by *Enterobacter gergoviae* and *Enterobacter ludwigii* at 10^6^ cells/mL, and by *Pseudomonas fulva* at 10^7^ cells/mL. *Ae. albopictus* was attracted by 6 of the 14 bacterial species, but each species was attractive at only a single cell density: *Bacillus thuringiensis*, *Lactococcus lactis*, *Enterobacter ludwigii*, *Pseudomonas plecoglossicida*, and *Citrobacter freundii* were attractive at 10^7^ cells/mL, while *Brevundimonas vesicularis* was attractive at 10^6^ cells/mL [53].

Microorganisms related to vertical transmission via eggs were shown to belong to *Chryseobacterium* and *Delftia*, two abundant genera in adult *Ae. aegypti* after a blood meal [26], and to the genera *Asaia* [26] and *Wolbachia* [54]. This is important in breeding containers for *Aedes* spp., as adult females can modify the microbial composition of small aquatic environments and, consequently, the composition of larval microbiota [37].

Larvae themselves can modify the microbial composition of their environment. Indeed, Kaufman et al., showed that, in water, *Ae. triseriatus* Say contributes to creating enriched and anoxic conditions that favor the growth of facultative anaerobes, such as Enterobacteriaceae [55].

### 2.2. Fungi

In addition to bacteria, microbiota is composed of fungal communities, even if only a few researchers have focused on the study of the fungal microbiota in mosquitoes [56,57]. Steyn et al., described the microbiota of *Culex pipiens* Linnaeus and *Cx. theileri* Theobald, identifying different genera including *Candida*, *Cryptococcus*, *Galactomyces*, *Hannaella*, *Meyerozyma*, *Pichia*, *Rhodosporidium*, *Rhodotorula*, *Trichosporon*, and *Wickerhamomyces* [57]. Bozic et al., showed that mosquitoes can host yeasts of clinical importance in their microbiota [56]. In the same study, the researchers also isolated the opportunistic pathogen *Candida parapsilosis* in several species, and at different stages of development, of laboratory-bred mosquitoes, namely *An. gambiae*, *An. stephensi*, *Culex quinquefasciatus* Say, *Ae. albopictus*, and *Ae. aegypti*. Furthermore, the analysis of the microbiota of wild *Cx. pipiens* and *Cx. theileri* at the larval stage revealed the presence of clinically important yeast species, including the well-known human pathogen *Candida albicans* [28].

### 2.3. Algae

Among the microorganisms that share the same habitat with mosquito larvae are several species of algae and microalgae (phytoplankton). In nature, these algae and microalgae are an important source of nutrition for the larvae of many mosquito species, as shown by the large quantity of algae found in their guts [58,59,60,61].

Charles et al., identified 55 different species of algae in the midgut of eight mosquito species [60]. Bond et al., showed that removing *Spyrogira* sp. from the habitat of *An. pseudopunctipennis* Theobald led to a decline both in larval and adult populations. This suggests the importance of *Spyrogira* for this mosquito species, either as a food source or as a place of refuge from predators [62]. However, other authors showed that some green algae species may be responsible for a decrease in larval populations due to their indigestibility [63]. Kiviranta and Abdel-Hameed showed how the *Oscillatoria* blue algae may be toxic to *Ae. aegypti* larvae [64].

### 2.4. Protozoa

Protozoa are omnipresent in aquatic environments and live in close contact with mosquito larvae, sharing the same habitat. These microorganisms appear to contribute to the larval development of mosquitoes, acting as intermediate consumers: the protozoa that feed on bacteria themselves become food for the mosquito larvae, providing a direct link in the transfer of bacterial biomass [65,66].

### 2.5. Viruses

Some years ago, a group of insect-specific viruses (IsV) were described [67] These viruses, unlike arboviruses, which can replicate in both mosquitoes and vertebrates, have insects as their only host and do not infect vertebrates. For mosquitoes, these viruses are called mosquito-specific viruses (MsV). The first MsVs identified were the cell-fusing agent virus (CFAV), Kamiti River virus (KRV), and *Culex* flavivirus (CxFV) [68,69,70]. In the last decade, thanks to next-generation sequencing techniques, many MsVs were described [27]. The possible role of these viruses in mosquito biology remains to be determined.

## 3. Microorganisms as Mosquito Larval Diet

Mosquito larvae are aquatic omnivorous animals that feed on a variety of particles [71]. Usually, mosquito breeders use diets rich in all the necessary nutrients to ensure good development from the larval to the adult stage [71,72]. Several studies have suggested how the amount and quality of food present in water may influence the development of larvae in nature [73,74]. In the laboratory, it was found that the diet of the larvae has a huge impact on their development and survival, as there is a minimum amount of nutrients needed to trigger cascading hormonal reactions necessary for development into the adult stage [75]. Elora and Sarkar showed that, in *Cx. quinquefasciatus*, larval growth was positively correlated with the quantity of carbohydrates and lipids in the diet, but it was negatively correlated with the protein content [76]. The highest percentage of adult yields were, however, obtained with protein-rich diets. The authors suggested that the effects of diet on the larvae could be mediated by differences in microbiota load and/or composition.

Souza et al., showed that specific biological parameters, such as pupation time, emergence, and survivorship, were lower when certain bacteria (*Serratia marcescens*, *Escherichia coli*, *Staphylococcus aureus*, *Bacillus* sp., *Asaia* sp., and *Ochrobactrum intermedium*) were used as the only *Ae. aegypti* larval food source than when a commonly utilized diet, such as TetraMin^®^ (Tetra Holding GmbH., Melle, Germany), a food for aquarium fish, was used [28].

Travanty et al., evaluated the effects of five bacteria species (*Porphyrobacter* sp., *Acidiphilium rubrum*, *Azorhizobium caulinodans*, *Enterobacter asburiae*, and *Pseudomonas syringae*), isolated from the leaf litter of *Quercus alba*, on the survival and development of *Ae. albopictus* larvae [20]. The effects of each bacterial species, of the five combined bacteria, and of a diverse microbial community from an infusion of litter were compared. Results showed that both individual bacteria and their combination did not guarantee adequate survival and development of the larvae. The results were different for the larvae fed with the litter microbial community, which showed efficient larval growth. Although an exclusively bacterial diet is not ideal for the development of mosquitoes, the enrichment of the diet with certain bacterial species has been shown to have beneficial effects [20].

Souza et al., evaluated different biological parameters, such as the sex ratio and the size of the wings in the two sexes of *Ae. aegypti* [28]. In all the tested diets based on microorganisms, there was an imbalance in the sex ratio in favor of the males. The same study also showed that a diet based on microorganisms could affect the wing size of adults and, probably, also their body size. Males and females fed with *E. coli* alone exhibited smaller wing sizes than males fed with *Asaia* sp. alone. Moreover, using yeast as a food source, Souza showed that males fed with *Pseudozyma* sp. or *Saccharomyces cerevisiae* had similar wing sizes, while females fed with *S. cerevisiae* had larger wings than those fed with *Pseudozyma* sp. The wings in both sexes were larger when the control diet (TetraMin^®^) was used than when a microorganism-based diet was used, even if *S. cerevisiae* gave results closer to those obtained using TetraMin^®^ [28].

The effects of algae used as a food source have also been studied by several authors. Ahmad et al., showed that *Ae. aegypti* larvae fed with the algae *Scenedesmus* sp. and *Chloroccocum* sp. had delayed development, and metamorphosed into smaller adults than those fed with the control diet (finely ground cooked liver) [77]. On the other hand, *Ae. aegypti* larvae fed with the algae *Ankistrodesmus convolutus* and *Chloroccocum* sp. attained a greater body size than those fed with the control diet. The data obtained suggested that *A. convolutus* and *Chlorococcum* sp. were an adequate source of food for the development of *Ae. aegypti* [77].

The hypothesis that protozoa, as a food source, may play a positive role in the development and survival of the larvae of different mosquito species is still being studied. Östman et al., noted the proliferation of different genera of protozoa after the elimination of the *Aedes* mosquito larvae using *Bacillus thuringiensis* ser. *israelensis* larvicide. This observation suggests a strong regulation of the protozoan population by *Aedes* larvae [78].

Skiff and Yee showed experimentally that *Ae. albopictus* and *Cx. coronator* had no measurable differences in performance and survival when fed with bacteria alone or with bacteria and protozoa (both conditions were tested with the addition of a mixture of lactalbumin-yeast), but different survival was observed between the two species with *Ae. albopictus* showing significantly higher survival than *Cx. coronator* [79]. In another experiment in the same study, three mosquito species, *Ae. albopictus, Cx. coronator,* and *Cx. quinquefasciatus*, were fed with only bacteria or fed with bacteria and protozoa (both conditions in the absence of lactalbumin–yeast mixture). The larval survival was different among the three species, although their feeding treatments were the same, thus suggesting that the nutritional role of protozoa in mosquito is still uncertain [79]. In the same experiment, it was shown that *Ae. albopictus* had longer survival than the two *Culex* species. This was probably due to the higher competitiveness of *Ae. albopictus*, which needs fewer resources to survive compared to *Culex* [80], rather than to a different effect of the feeding treatments on *Ae. albopictus* compared to *Culex*.

In another work, Duguma et al., measured larval development, emergence, and adult biomass of *Cx. nigripalpus* in aquatic containers integrated with ciliated protozoa and rotifers. The results showed that the addition of ciliates and rotifers altered the resources available for mosquito larvae and, consequently, their development [81]. The authors suggested that ciliated protozoa and rotifers did not serve as food for the young mosquito larvae, but rather competed with them for the acquisition of microbial biomass [82].

Although it now seems certain and well-established that in nature mosquito larvae feed on various microorganisms, from laboratory observations, these microorganisms do not appear to be advantageous to their development [28]. The above studies, however, focused almost exclusively on the effects of microorganisms as the only food source in larval diets, failing to investigate their possible effects as probiotics.

## 4. The Potential Use of Probiotics in Diet

Given the importance of microbiota in regulating various host functions, a promising research path may be the study of the integration of probiotics into diets, with the aim of improving some useful characteristics, i.e., larval survival and adult quality parameters such as longevity, dispersal, and male sexual performance. Mitraka et al., showed how a larval diet enriched with *Asaia* sp. shortened the larval development time [82]. Guégan et al., claimed that *Asaia* sp. contributed to larval development in *An. stephensi*, since it was found to be closely associated with this mosquito [27]. These findings suggest that *Asaia* sp. may play a positive role in the development of mosquitoes.

Souza et al., showed that the longevity of *Ae. aegypti* fed with *Asaia sp.* and *E. coli* was higher than the longevity obtained using other bacteria as a food source. These results suggest that *E. coli* and *Asaia* sp. may be beneficial to mosquitoes [28].

Although in the literature no studies were found on the addition of probiotics in the diet of mosquitoes used for SIT programs, some research has focused on the use of probiotics to improve the male performance of other insect species.

Heithem Hamden et al., showed that, by enriching the diet of the Mediterranean fruit fly, *Ceratitis capitata* Wiedemann, with various bacterial species (*Klebsiella pneumoniae*, *Enterobacter* spp. and *Citrobacter freundii*), there was a change in the insect’s microbiota components, which led to an improvement in the quality control parameters of the males (i.e., increase in pupal weight, adult emergence, longevity, adult size, and flight ability) [83].

Augustinos et al., used one of the bacterial species (*Enterobacter* spp.) identified in *C. capitata* microbiota as a probiotic in the larval diet. The results showed an improvement in the productivity of pupae and adults, and a shortening of the development time of the immature stages, especially for males [24].

Yao et al., and Shuttleworth et al., used a strain of *Enterobacter* as a probiotic to supplement the larval diet of the melon fly, *Zeogodacus* (=*Bactrocera*) *cucurbitae* (Coquillet), leading to a significant increase in pupal size, adults, and survival rate [83,84].

Shuttleworth and collaborators tested different bacteria present in the microbiota of the Queensland fruit fly, *Bactrocera tryoni* (Froggatt), as supplements to the larval diet, with interesting results. In particular, they noticed that, when the diet was supplemented with *Enterobacter* sp. and *Asaia* sp., the time for larval development decreased, while it increased when strains of *Lactobacillus* sp., *Leuconostoc* sp., and a mix of them were used [85]. However, they also found that the total development time (from egg hatching to adult emergence) was reduced by supplementing the larval diet with *Lactobacillus* sp., *Leuconostoc* sp., and a mix of them, compared to when *Enterobacter* sp. and *Asaia* sp were used. This result is important because a shorter time of development could boost productivity in a mass rearing context, making it possible to produce a greater number of insects within the same amount of time [85]. Moreover, the same authors noticed that the integration of bacteria into the larval diet did not lead to an increase in the pupal size, rate of emergence, better flight skills, or an imbalance of the *sex ratio* in *B. tryoni* [85].

## 5. Conclusions

Based on the small amount of data found in the literature, we hypothesize that a better understanding of the interaction between larval diet and microbiota may bring significant improvements in mosquito mass rearing for SIT purposes.

Studies have suggested that the exclusive use of microorganisms as larval nutriment is inefficient due to their poor nutritional supply compared to commonly used standard diets, such as TetraMin^®^ [28]. Several authors have shown improved larval development connected with the interaction between diet and microorganisms [23,24,82]. *Escherichia coli* and *Asaia* sp. could be interesting candidates for evaluating the possible application of microorganisms in mosquito larval breeding. In addition, the bacteria *Dysgonomonas* spp., which were found to be abundant in *Ae. albopictus* and able to produce vitamin B12 in termites, may deserve more investigation [35].

Although microorganisms that may be pathogenic to humans have been identified in the microbiota of mosquitoes [56], these are not used as probiotics. However, for worker safety, it remains important to wear Personal Protective Equipment (PPE).

Although some studies have already shown how the enrichment of larval diet with microorganisms could improve sterile male production, further research is needed to confirm these findings. For example, Ben Ami et al., suggested that the exposition of *C. capitata* pupae to gamma rays used for SIT could compromise the microbiota composition in the target insect [86]. This phenomenon was also observed by Lauzon and Potter in the males of *C. capitata* and *Anastrepha ludens* Loew (Mexican fruit fly), and led to damaged intestinal tissue, intestinal cell organelle, and intestinal microbiota. This phenomenon was not observed in non-irradiated specimens of the same age [87].

This aspect could be a problem, considering that the relationship between many insect species and their microbiota is mutualistic, and microorganisms could contribute to the host fitness [88,89]. Another study on *C. capitata* showed that probiotic addition to the adult glucophage diet improved the sterile male competitiveness [89]. This could be due to the effect of the microbiota restored by the probiotics.

A possible strategy could be to integrate the diet of both larvae and adult males with probiotics, in order to improve both the breeding conditions of the pre-imaginal stages and the competitiveness of the males [87,88].

Another interesting aspect is the role certain bacteria play in oviposition. Ponnusamy et al., identified nine bacteria in *Ae. aegypti* and six in *Ae. albopictus* that, at certain densities, were capable of inducing greater oviposition than the control medium [53]. The reason why certain microorganisms, at certain densities, favor oviposition while, at higher densities, they inhibit it, has been proposed by Ponnussamy and colleagues. They observed that different bacteria produced substances, such as certain carboxylic acids and specific esters, that radically modified the choice of the deposition site of gravid *Ae. aegypti* [90]. They found that certain substances associated with these bacteria induced oviposition at specific concentrations, while the same substances at higher concentrations (e.g., tetradecanoic acid), or other signals produced by the same or different bacteria (e.g., methyl ester hexadecanoic acid), discouraged egg laying by gravid females [90]. The fact that, with change in the microbial density, the same bacterial species can favor or discourage oviposition can lead to an approach where certain microorganisms are used to increase the number of eggs laid in a mass rearing context.

Good quality of sterile males is a key tool in SIT programs. Sterile male quality is a complex subject whose definition derives from a combination of several parameters that need to be optimized, such as male longevity in harsh conditions, resistance to stressful conditions, dispersal propensity, and mating competitiveness for wild females [91,92,93]. Production of large quantities of high-quality sterile males that are able to compete favorably with wild males would result in greater efficiency of a SIT program and, therefore, improve the cost–benefit balance.

Subsequent studies should focus on the integration of probiotics into the larval as well as the adult diets, checking different parameters representative of good rearing conditions, such as pupation dynamics, mortality, and male productivity. To test the beneficial effects of microorganisms, it is necessary to develop proper quality control methodologies aimed at assessing flight ability, longevity, dispersion, and field competitiveness.

In conclusion, considering all the above-mentioned aspects, we can argue that, by optimizing the microbiota composition during larval rearing and in the adult stage, the efficiency of genetic mosquito control methods requiring mass rearing could be improved.
